# The effectiveness of interventions to prevent loneliness and social isolation in the community-dwelling and old population: an overview of systematic reviews and meta-analysis

**DOI:** 10.1093/eurpub/ckad006

**Published:** 2023-03-09

**Authors:** Ludwig Grillich, Viktoria Titscher, Pauline Klingenstein, Eva Kostial, Robert Emprechtinger, Irma Klerings, Isolde Sommer, Jana Nikitin, Anton-Rupert Laireiter

**Affiliations:** Department for Evidence-Based Medicine and Evaluation, University for Continuing Education Krems, Krems an der Donau, Austria; Department of Clinical and Health Psychology, Faculty of Psychology, University of Vienna, Vienna, Austria; Department for Evidence-Based Medicine and Evaluation, University for Continuing Education Krems, Krems an der Donau, Austria; Department for Evidence-Based Medicine and Evaluation, University for Continuing Education Krems, Krems an der Donau, Austria; “Tut gut!” Gesundheitsvorsorge GmbH, Sankt Pölten, Austria; Faculty for Health and Medicine, University for Continuing Education Krems, Krems an der Donau, Austria; Department for Evidence-Based Medicine and Evaluation, University for Continuing Education Krems, Krems an der Donau, Austria; Department for Evidence-Based Medicine and Evaluation, University for Continuing Education Krems, Krems an der Donau, Austria; Department of Development and Educational Psychology, Faculty of Psychology, University of Vienna, Vienna, Austria; Department of Clinical and Health Psychology, Faculty of Psychology, University of Vienna, Vienna, Austria; Department for Psychology, Paris Lodron University of Salzburg, Salzburg, Austria

**Keywords:** Loneliness, social isolation, older adults, review of reviews

## Abstract

**Background:**

Loneliness and social isolation have comparable health effects to widely acknowledged and established risk factors. Although old people are particularly affected, the effectiveness of interventions to prevent and/or mitigate social isolation and loneliness in the community-dwelling older adults is unclear. The aim of this review of reviews was to pool the findings of systematic reviews (SRs) addressing the question of effectiveness.

**Methods:**

Ovid MEDLINE^®^, Health Evidence, Epistemonikos and Global Health (EBSCO) were searched from January 2017 to November 2021. Two reviewers independently assessed each SR in two consecutive steps based on previously defined eligibility criteria and appraised the methodological quality using a measurement tool to assess SRs 2, AMSTAR 2. One author extracted data from both SRs and eligible studies; another checked this. We conducted meta-analyses to pool the study results. We report the results of the random-effects and common-effect models.

**Results:**

We identified five SRs containing a total of 30 eligible studies, 16 with a low or moderate risk of bias. Our random-effects meta-analysis indicates an overall SMD effect of 0.63 [95% confidence interval (CI): −0.10 to 1.36] for loneliness and was unable to detect an overall effect of the interventions on social support [SMD: 0.00; 95% CI: −0.11 to 0.12].

**Discussion:**

The results show interventions can potentially reduce loneliness in the non-institutionalized, community-dwelling and older population living at home. As confidence in the evidence is low, rigorous evaluation is recommended.

**Registration:**

International Prospective Register of SRs (PROSPERO): Registration number: CRD42021255625

## Introduction

Social isolation and loneliness have comparable health effects to widely acknowledged and established risk factors, such as smoking, physical inactivity, obesity and hypertension. They have been found to increase the risk of cardiovascular disease by 27%^1^ and the risk of mortality by 26%.^2^

The available literature shows a wide variation in the prevalence of loneliness for older adults (60+ years). With northern European countries having the lowest prevalence (5.2%) and eastern European countries having the highest prevalence of loneliness (21.3%).^3^

While everyone is at risk of experiencing loneliness to some degree at different points in their lives, the risk of experiencing events that can trigger social isolation and loneliness increases with age due to personal losses and, consequently, a decrease in network size and activity levels.^4^ Retirement from work corresponds to a loss of meaningful social contacts, and with increasing age comes an increased risk of loss of mobility, deaths of relatives and friends and a reduction of social network size.^5^

Considering the rising life expectancy and the proportional increase of people over 60,^6^ social isolation and loneliness are, therefore, an increasingly important health challenge for societies worldwide. Taking into consideration that social distancing measures were implemented to reduce the risk of infections during the SARS-CoV-2 pandemic, we must acknowledge that these measures led to an increase in social isolation and loneliness, with old people affected most severely.^7^

Thus, there is a growing scientific, public and political interest in interventions to prevent loneliness and social isolation or to mitigate their effects in older adults.^8^ For evidence-based practice, the selection of interventions should be based on the best evidence of the desired effects.^9^

Systematic reviews (SRs) are the most appropriate method to guide the selection of appropriate interventions in public health, as they reduce bias in the synthesis of the best available evidence. However, healthcare providers wanting to prevent social isolation and loneliness among old people are confronted with a great number of such SRs. The first review on this topic was published by Rook in 1984^10^ and was followed by three more reviews until 2003 and by at least one additional review every year since 2010.^4^ To further complicate matters, the SRs published to date are of differing quality and vary in their conceptual understanding of social isolation and loneliness.^11^ Therefore, healthcare providers need a method to make sense of this inconsistent information. Overviews of SRs—also called umbrella reviews, meta-reviews, reviews of reviews or reviews of meta-analyses—offer a solution to this challenge.^12^ They summarize evidence from more than one SR of different interventions for the same condition or problem,^13^ compile their results according to a predefined procedure, assess their quality and summarize their evidence for relevant endpoints.^14^

As most of the old people affected by the risk of social isolation and loneliness reside in the community, the focus of this overview of reviews will be on community-dwelling adults over the age of 60 living at home.

In the context of this article, we will define ‘social isolation’ as an objective state of having little or no contact with one’s personal social networks, namely families, friends and communities.^15^ ‘Loneliness’ we understand as a distressful subjective experience associated with realizing that one’s needs for social affiliation and bonding are not met given the quality or quantity of current relationships.^16^ Accordingly, there may be people who have many social contacts but subjectively feel lonely, while there may be people who lack social company and conversely do not feel lonely. Due to this, it is imperative to examine how social isolation and loneliness are defined and measured when evaluating the literature.^17^

To the best of our knowledge, this is the first review of reviews addressing the question of the effectiveness of interventions to prevent and/or mitigate social isolation and loneliness in the non-institutionalized, independently living, community-dwelling old people.

## Methods

We registered the protocol of this review of reviews at the International Prospective Register of SRs (PROSPERO): Registration number: CRD42021255625. We adhered to the Preferred Reporting Items for SRs and Meta-Analyses (PRISMA) statement^18^ throughout this manuscript (PRISMA checklist, see Supplementary appendix A).

### Study design

We conducted an overview of reviews following the guidance provided in the Cochrane Handbook.^19^

### Search strategy

The full search strategy is presented in Supplementary appendix B. An information specialist searched Ovid MEDLINE^®^, Health Evidence, Epistemonikos and Global Health (EBSCO). The usefulness of SRs also depends on their actuality, but there is no consensus on when SRs are obsolete and when an update is necessary.^20^ To prevent us from relying on outdated evidence, we limited the search to SRs published from 2017 onwards. The searches were conducted in November 2021 and were limited to seniors and SRs, depending on the database.

### Eligibility criteria

We predefined criteria to include or exclude SRs. Based on these criteria, we included all SRs of preventive or health-promoting interventions designed to prevent or mitigate social isolation/loneliness among independently living, community-dwelling old people. We excluded exclusively online-based interventions without face-to-face contact. For a detailed description of the inclusion and exclusion criteria, see Supplementary appendix C.

We defined SR according to the Cochrane Handbook^21^ and ‘interventions’ as a ‘coordinated sets of activities designed to change specified behaviour patterns’.^22^

### SR selection

The reviewer team consisted of three people with experience in conducting SRs (L.G., V.T. and I.S.). Two reviewers (L.G. and V.T.) independently assessed each SR in two consecutive steps (abstract and full-text selection) based on the previously defined eligibility criteria. They resolved any discrepancies by discussion or consulting a third reviewer. We used Covidence software (https://www.covidence.org/) for the SR selection process.

### Risk of bias assessment and certainty of evidence

Three reviewers (L.G., P.K. and V.T.) appraised the methodological quality of the SRs with a measurement tool to assess SRs 2 (AMSTAR 2).^23^ This tool provides an assessment of the overall confidence in the results of each review using the following categories: critically low, low, moderate or high (for an interpretation of these categories, see Supplementary appendix D). Disagreements in appraisal between the reviewers were solved through discussion and consensus. The quality assessment of the primary studies was conducted by the authors of the SR and was further used for this overview of reviews.

### Study selection

The included SRs also reported on studies that did not meet our pre-specified eligibility criteria (see Supplementary appendix C). To draw valid conclusions to answer the research question, it was, therefore, necessary to select the studies reported in the included reviews. We did this using the same eligibility criteria we used to select the syntheses. One author (P.K.) reviewed the studies included in the SR to see if they met the eligibility criteria and a second author (L.G.) checked this.

### Data extraction

We only included SRs with a low, moderate or high confidence in the results and extracted data from both the SR and included studies.

For all SRs, we extracted the following data: SR details (author, title, year of publication and aim of the SR); included studies’ details (number and type of studies and persons included, risk of bias tool used and risk of bias of studies); population details (age, gender and health status); intervention details (duration and type of intervention) and result details (outcomes and measuring instruments).

For all studies reported in the SRs that met the eligibility criteria, we extracted the following data and supplemented them from the primary studies as needed: study details (author, title, year of publication, study type and number of participants); participants’ details (country, age, gender, residence status, pre-existing condition and subgroup); intervention details (short description, type of intervention, format of intervention, kind of staff, duration, frequency, intensity and setting) and outcome details (outcome type, measurement tool, direction of effect for all outcomes, effect size for outcomes addressing social isolation and loneliness).

### Synthesis and meta-analysis

As there is little confidence in the results of high-risk studies, for all outcomes measuring loneliness and social isolation, we synthesized the data of the studies with a low and/or moderate risk of bias. We conducted meta-analyses to pool the study results. We reported the results of the random-effects and common-effect models. The between-study variance was estimated with restricted maximum likelihood. We extracted mean values and their associated standard deviations at the last follow-up. In case the studies did not report these outcomes, we either tried to calculate or approximate them using other outcomes. The specific approach for each individual outcome can be found in the Supplementary appendix. We conducted all analyses in the R environment (Version 4.1.1) using the meta and tidyverse packages. We categorized the interventions according to thematic analysis and the previous literature in this field.^24^^,^^25^

There is a variability in the existing measurement tools for social isolation and loneliness. Valtorta *et al.*^11^ identified 54 measurement instruments and found that ‘tools explicitly designed for measuring loneliness … tend to be based on more subjective questions, whereas social network indices primarily use more objective measures’ (p. 6). In our analysis, we followed these findings and grouped the outcomes according to the instruments used in the primary studies.

## Results

**Figure 1:**
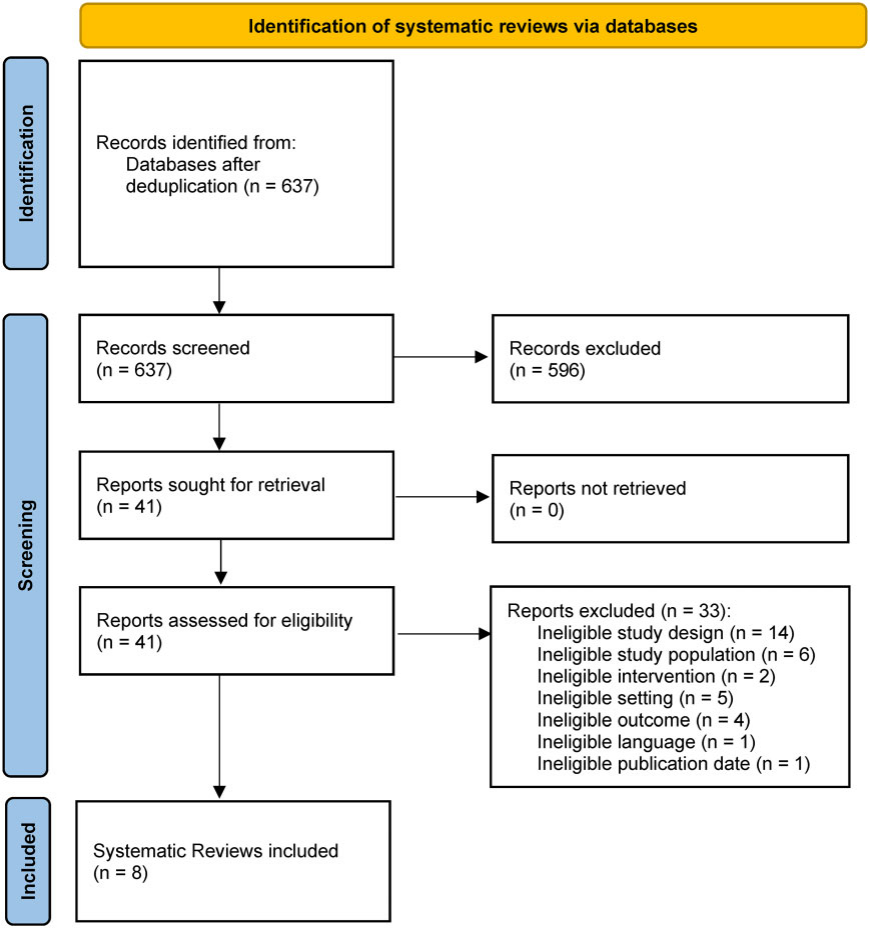
PRISMA flow chart

Figure 1 summarizes the results of the search. We identified 637 citations after removal of duplicates. Three authors (L.G., P.K. and V.T.) independently screened all abstracts and 41 full texts against the pre-specified eligibility criteria. We included eight reviews.^26–33^

### Description of the included reviews

Table 1 shows the main characteristics of the included SRs (see Supplementary appendix E for further details). The included SRs were published between 1985 and 2021 and reported on about 176 studies (including duplicates). All SRs reported about the effectiveness of different intervention types on loneliness and/or social isolation. However, due to a lack of available data, none of these SRs conducted a meta-analysis on loneliness or social isolation. The quality of the SRs was moderate for four SR,^26^^,^^29–31^ low for two^27^^,^^28^ and critically low for two.^27^^,^^32^ The following critical domains were the most common reasons for downgrading: (i) missing a statement that the review methods were established prior to the conduct of the review and (ii) lack of consideration of the risk of bias of individual studies when interpreting/discussing the results and lack of a list of excluded studies to justify the exclusion. To assess the risk of bias of the included SRs, four SRs^26^^,^^27^^,^^29^^,^^32^ used the Cochrane Risk of Bias Tool,^34^ three^28^^,^^31^ used the Effective Public Health Practice Project tool,^35^ one^30^ the U.S. Preventive Services Task Force criteria^36^ and one the Joanna Briggs Appraisal Checklist.^37^ For the characteristics of the included reviews, see table 1.

**Table 1:** Characteristics of the included reviews

Review reference	Year	Stated aim of the review	Number and type of studies included	Population	Quality assessment
Coll-Planas et al.^26^	2017	‘Assessing the impact on health outcomes and use of health-related resources of interventions that promote social capital or its components among older people’.	36 RCTs	Mainly Caucasian older people without disability or dementia	Moderate
Heins et al.^31^	2021	‘To provide a systematic overview of the effects of technological interventions that target social participation in community-dwelling older adults with and without dementia’.	36 (6 RCT, 4 nRCT, 10 pre-post, 2 post, 14 qualitative)	Community-dwelling older adults with and without cognitive impairment	Moderate
Li et al.^27^	2018	‘With the aim of synthesizing the existing literature and to provide implications for improving social well-being in older adults using exergaming’.	10 (5 nRCTs, 5 qualitative)	Mainly Caucasian healthy older adults	Low
Manjunath et al.^33^	2021	‘To assess the quality of the studies and to identify effective recommendations for interventions against social isolation’.	20 (13 nRCT, 7 pre-post)	Older adults experiencing loneliness	Critically low
Poscia et al.^28^	2018	‘To summarize and update the current knowledge on the effectiveness of the existing interventions for alleviating loneliness and social isolation among older persons’.	20 (4 RCTs, 3 nRCTs, 6 pre-post, 3 post-test, 2 qualitative)	Mainly Caucasian community-dwelling older adults and those from home care centres	Low
Shvedko et al.^29^	2018	‘To examine physical activity intervention effects on loneliness, social isolation or low social support in community-dwelling older adults’.	38 RCTs	Caucasian older people; two-third of the studies included people with chronic diseases, only one-third with healthy people	Moderate
Tong et al.^32^	2021	‘To summarize and update the current knowledge about the efficacy of existing interventions for alleviating social isolation and loneliness among older adults’.	24 RCTs	Mainly Caucasian older adults	Critically low
Veazie et al.^30^	2019	‘Effectiveness of interventions that target social isolation and loneliness to improve health and reduce unnecessary health care utilization and -harms associated with interventions’.	16 (7 RCTs, 8 pre-post, 1 post)	Mainly Caucasian healthy, community-dwelling older adults	Moderate

### Description of the included studies

Thirty-six studies^40–75^ published between 1985 and 2019 reported in the included SRs met the eligibility criteria (see additional reference list in Supplementary appendix J): six non-randomized controlled trials (nRCTs),^40,56,57,59,68,72^ one post,^62^ 11 pre-post^42,46,47,49,51,55,63,64,71,74,75^ and 18 randomized controlled trials (RCTs).^41,43–45,48,50,52–54,58,60,61,65–67,69,70,73^ According to the SR, the risk of bias was high for 19 studies,^41–43,46,47,49–51,55,58,59,62–64,67,68,71,74,75^ moderate for 10^40,44,48,56,57,65,69,70,72,73^ and low for 7.^45,52–54,60,61,66^

### Characteristics of the participants

At least 4243 people in total took part in the studies. The mean age of the participants was between 58 and 83, and the majority of participants were female, at an average percentage of 70%. For details, see Supplementary appendix E.

### Characteristics of the interventions

We grouped the interventions into seven types: art, education, physical activity, social support training and multicomponent (combining more than one type of intervention). The majority of studies examined multicomponent interventions^42–44,48,51,52,54,56–58,60,62,65–68,70–75^ followed by social support^40–42,46,47,49,50,61,63^ and physical activity interventions.^45,53,54,58^ For details, see Supplementary appendix F.

### Outcomes

In our analysis, we follow the findings of Valtorta *et al.*^11^ and combined the outcomes reported in the studies according to the measurement tool used into two central outcomes: loneliness and social isolation/support. We assigned all outcomes measured with the following measurement tools to loneliness (see additional reference list in Supplementary appendix J): the de Jong Gierveld Loneliness Scale,^76^ the UCLA Loneliness Scale^77^ and a three-item loneliness scale.^78^ We assigned all outcomes measured with the following measurement tools to social isolation/support: Adaptation of The Duke Social Support Index,^78^ Inventory of Social Supportive Behaviours,^79^ Lubben Social Network Scale,^80^ Multidimensional Scale of Perceived Social Support,^81^ Oslo-3 Social Support Scale,^82^ Revised Social Support Questionnaire,^83^ Social Support List Interactions,^84^ Social Support Survey,^85^ Perceived Isolation Scale,^86^ MOS Social Support Survey^85^ and the Friendship Scale.^87^

### Loneliness

Loneliness is a distressful subjective experience associated with realizing that one’s needs for social affiliation and bonding are not met given the quality or quantity of actual relationships.^16^

We identified 22 studies^40–42,44,46–51,55–57,59,61,65–68,72–74^ in 4 SRs^26–28,30,31^ that measured loneliness. The risk of bias was high for 12 studies,^41,42,46,47,49–51,55,59,67,68,74^ moderate for 8^40,44,48,56,57,65,72,73^ and low for 2.^61,66^

Out of the 10 studies with a low or moderate risk of bias, 6^40,44,56,57,61,73^ showed an effect and 4^48,65,66,72^ did not (for details, see Supplementary appendix G). All the studies that showed an effect were group or mixed interventions. Four^44,56,57,73^ were multicomponent interventions and two^40,61^ were social support interventions. Four were conducted in Asia,^40,44,56,73^ one in the USA^57^ and one in the UK.^61^

**Figure 2:**
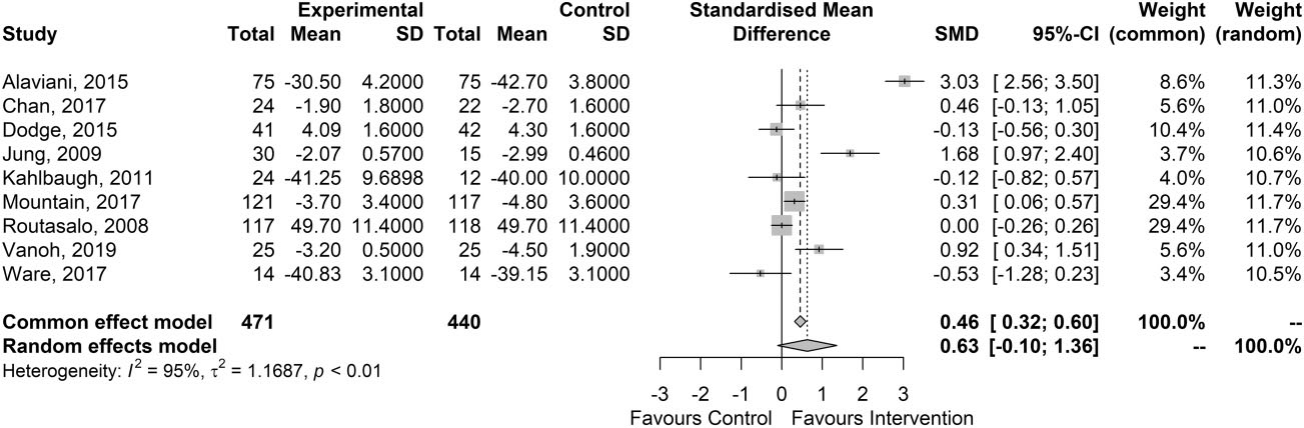
Forest plot of the included studies on the effects on loneliness

Our random-effects meta-analysis indicated a mean overall effect of SMD 0.63 favouring the interventions [95% confidence interval (CI): −0.10 to 1.36] (see figure 2).

### Social isolation/support

In the context of this article, we define ‘social isolation’ as an objective state of having little or no contact with one’s personal social networks, that is, families, friends and communities^15^ and ‘social support’ as one’s social network.^17^

We identified 24 studies^41–46,51–55,58,60,62–64,66–71,73,75^ that measured social isolation/support. The risk of bias was high for 14 studies,^41–43,46,51,55,58,62–64,67,68,71,75^ moderate for 4^44,69,70,73^ and low for 6.^45,52–54,60,66^ None of the studies with a low or moderate risk of bias showed an effect. For details, see Supplementary appendix H.

**Figure 3:**
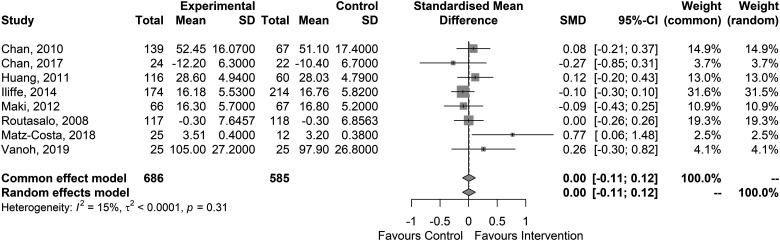
Forest plot of the included studies on the effects on social isolation/support

The meta-analysis was unable to detect an overall effect of the interventions on social support [SMD: 0.00; 95% CI: −0.11 to 0.12] (see figure 3).

Details of the meta-analysis can be found in Supplementary appendix I.

## Discussion

This overview of reviews provides a comprehensive summary of the effectiveness of interventions to prevent and/or mitigate the effects of social isolation and loneliness in the non-institutionalized, community-dwelling old people. We identified SRs that assessed the effectiveness of these interventions in general^28^^,^^30^^,^^32^^,^^33^ and SRs that assessed specific interventions: promoting social capital,^26^ digital gaming and physical exercise,27 technological interventions^31^ and physical activity.29 None of these SRs conducted a meta-analysis on the outcomes addressing aspects of social relationships.

Two^27^^,^^28^ of the five included SRs were of low, two^32^^,^^33^ of critically low quality and therefore may not provide an accurate and comprehensive summary of the available studies.

All SRs focused on older people in different living conditions (institutionalized, living at home) and considered both health-related outcomes (mental health, physical health and function and quality of life) and outcomes related to interpersonal relationships such as loneliness and social isolation/support. The SRs did not differentiate between different groups of patients and outcomes in their conclusions.

Over half of the included studies had a high risk of bias and their results were thus unreliable. This demonstrates that there is also a need for more and better-quality studies on this topic. To make reliable statements about effectiveness, we therefore omitted studies with a high risk of bias.

We observed a great variety of different measuring instruments and techniques, and differences in how studies report outcomes. Although loneliness and social isolation are different concepts,^11^ studies used them interchangeably. Part of this variability in measurement is probably the reason for the different results and the contradictory conclusions of the syntheses. Therefore, we did not group the results according to how the authors defined social isolation and loneliness, but according to the measurement instruments used.

The meta-analysis could not detect an overall effect of the interventions on social isolation/support but signs for an effect on loneliness (SMD: 0.63; 95% CI: [−0.10 to 1.36]). The SMD hints at a potential medium effect, but the large confidence interval indicates a high degree of uncertainty in this result. This suggests that further studies are needed to gain a more accurate understanding of the situation. The high statistical heterogeneity (*I*^2 = 95%) suggests that the effect depends on the methods used in the individual studies. Due the limited number of primary studies, we are not able to provide a meta-analytical assessment of what contributes to better or worse efficacy.

It may be that it is easier to change the subjective feeling of loneliness through interventions than to strengthen the social networks that contribute to social participation/isolation. Single interventions may not be sufficient to change social networks. If this is the case, measures may be needed that focus less on changing individual behaviours and more on strengthening existing and creating new social networks.

A recent meta-analysis showed the effectiveness of psychological interventions for loneliness.^38^ Yet, this meta-analysis includes psychological interventions in all settings and for all age groups, in contrast to our review which focuses exclusively on community-dwelling and older people. Furthermore, we could not identify any promising features of the interventions to reduce loneliness. The nature of the interventions varied greatly and made direct comparison very difficult. In addition, many studies inadequately report these intervention characteristics. This is in line with the literature, for example, a SR by Hoffmann *et al.*^39^ using 137 interventions from 133 studies with non-drug interventions shows that only 39% of the interventions were adequately described in the primary source or in the references, appendices or websites.

Our overview of reviews has several limitations. Despite a strong contextual influence on ‘loneliness intervention’ regarding the implementation and outcomes, none of the SRs reported about contextual aspects. For the sake of time, we excluded qualitative studies, which made it difficult to explain the mechanism by which these interventions addressed social isolation (e.g. through improving relationships, increasing access to health services and improving patients’ self-efficacy). We recognize that by limiting our population to only community-dwelling adults, we may have missed some old people who are most likely to have social isolation, such as those in long-term care.

There are several strengths of this review. Three researchers independently implemented all the essential steps of this review of reviews, which minimized subjective influence in the screening and assessment. Another strength of the study is the precise conceptual distinction between social isolation and loneliness based on the measurement tools used. Finally, the meta-analysis enables a quantitative statement about the effectiveness of interventions to prevent and/or mitigate social isolation and loneliness in the non-institutionalized, independently living, community-dwelling old people.

The results show that interventions have the potential to reduce loneliness in the non-institutionalized, independently living, community-dwelling old people. However, confidence in the evidence is low. Therefore, we recommend that healthcare providers only implement interventions that are, firstly, based on a sound model of effectiveness and, secondly, subject to rigorous evaluation.

## Supplementary Material

ckad006_Supplementary_Data

## Data Availability

The essential data underlying this article are available in the article and in its online supplementary material. Further data will be disclosed to the corresponding author upon justified request.
