# Supplementary material for: The effectiveness of interventions to prevent loneliness and social isolation in the community-dwelling and old population: an overview of systematic reviews and meta-analysis
Source: Eur J Public Health. 2023 Mar 9;33(2):235–41. doi: 10.1093/eurpub/ckad006 (PMC10263264; doi:10.1093/eurpub/ckad006)
Supplement: ckad006_Supplementary_Data [file ckad006_Supplementary_Data.zip › ckad006_Supplementary_Data/ejph-2022-04-om-0208-File006.docx]

## Appendix B Search strategy

Ovid MEDLINE(R) ALL 1946 to November 12, 2021

| **#** | **Searches** |
| --- | --- |
| 1 | social isolation/ or loneliness/ |
| 2 | (social* adj1 isolat*).ti,ab,kf. |
| 3 | loneliness.ti,ab,kf. |
| 4 | lonely.ti,ab,kf. |
| 5 | or/1-4 |
| 6 | exp Aged/ or exp geriatrics/ or exp geriatric psychiatry/ or exp health services for the aged/ |
| 7 | (old* or aged or senior?).ti,kf. |
| 8 | ((old* or senior or aged) adj (population? or person? or people or men or women or adult? or patient?)).ab. |
| 9 | old age.ab. |
| 10 | (seniors or geriatr* or elder*).ti,ab,kf. |
| 11 | or/6-10 |
| 12 | 5 and 11 |
| 13 | limit 12 to "humans only (removes records about animals)" |
| 14 | Systematic Review.pt. |
| 15 | review.pt. |
| 16 | (medline or medlars or embase or pubmed or cochrane or (scisearch or psychinfo or psycinfo) or (psychlit or psyclit) or cinahl or ((hand adj2 search$) or (manual$ adj2 search$)) or (electronic database$ or bibliographic database$ or computeri?ed database$ or online database$) or (pooling or pooled or mantel haenszel) or (peto or dersimonian or der simonian or fixed effect)).tw,sh. or (retraction of publication or retracted publication).pt. |
| 17 | 15 and 16 |
| 18 | meta-analysis.pt. or meta-analysis.sh. or (meta-analys$ or meta analys$ or metaanalys$).tw,sh. or (systematic$ adj5 review$).tw,sh. or (systematic$ adj5 overview$).tw,sh. or (quantitativ$ adj5 review$).tw,sh. or (quantitativ$ adj5 overview$).tw,sh. or (quantitativ$ adj5 synthesis$).tw,sh. or (methodologic$ adj5 review$).tw,sh. or (methodologic$ adj5 overview$).tw,sh. or (integrative research review$ or research integration).tw. |
| 19 | ((scoping or rapid or mapping or umbrella) adj2 review).tw. |
| 20 | ((overview adj2 reviews) or "review of reviews").tw. |
| 21 | review?.ti. /freq=2 |
| 22 | 14 or 17 or 18 or 19 or 20 or 21 |
| 23 | 13 and 22 |
| 24 | limit 23 to yr="2017 -Current" |

Health Evidence (<https://www.healthevidence.org>) 15.11.2021

| Searches |
| --- |
| [("social isolation") OR loneliness] AND Limit:  Date = Published from 2017 to 2021 |
| Isolation AND Limit:  Date = Published from 2017 to 2021  Population = Seniors (60+ years) |

Epistemonikos (<https://www.epistemonikos.org>) 15.11.2021

| Search |
| --- |
| ((social* AND isolat*) OR loneliness OR lonely) AND (old* OR elder* OR aged OR senior OR seniors OR geriatr*) |
| Publication Year: 2017-2021 |
| Publication Types: Systematic Review, Broad Synthesis |

Global Health (EBSCO) 15.11.2021

| # | Query | Limiters/Expanders |
| --- | --- | --- |
| S1 | social* N1 isolat* | Expanders - Apply equivalent subjects |
|  |  | Search modes - Boolean/Phrase |
|  |  |  |
| S2 | loneliness | Expanders - Apply equivalent subjects |
|  |  | Search modes - Boolean/Phrase |
|  |  |  |
| S3 | lonely | Expanders - Apply equivalent subjects |
|  |  | Search modes - Boolean/Phrase |
|  |  |  |
| S4 | S1 OR S2 OR S3 | Expanders - Apply equivalent subjects |
|  |  | Search modes - Boolean/Phrase |
|  |  |  |
| S5 | systematic OR metaanaly* OR meta analy* | Expanders - Apply equivalent subjects |
|  |  | Search modes - Boolean/Phrase |
|  |  |  |
| S6 | (scoping OR rapid OR mapping OR evidence OR umbrella) N2 (review# OR synthe*) | Expanders - Apply equivalent subjects |
|  |  | Search modes - Boolean/Phrase |
|  |  |  |
| S7 | "overview of reviews" OR "review of reviews" | Expanders - Apply equivalent subjects |
|  |  | Search modes - Boolean/Phrase |
|  |  |  |
| S8 | S5 OR S6 OR S7 | Expanders - Apply equivalent subjects |
|  |  | Search modes - Boolean/Phrase |
|  |  |  |
| S9 | S4 AND S8 | Expanders - Apply equivalent subjects |
|  |  | Search modes - Boolean/Phrase |
|  |  |  |
| S10 | S9 | Limiters - Publication Year: 2017-2021 |
|  |  | Expanders - Apply equivalent subjects |
|  |  | Search modes - Boolean/Phrase |
