# Supplementary material for: The effectiveness of interventions to prevent loneliness and social isolation in the community-dwelling and old population: an overview of systematic reviews and meta-analysis
Source: Eur J Public Health. 2023 Mar 9;33(2):235–41. doi: 10.1093/eurpub/ckad006 (PMC10263264; doi:10.1093/eurpub/ckad006)
Supplement: ckad006_Supplementary_Data [file ckad006_Supplementary_Data.zip › ckad006_Supplementary_Data/ejph-2022-04-om-0208-File007.docx]

## Appendix C Eligibility criteria

|  | Inclusion criteria | Exclusion criteria |
| --- | --- | --- |
| Study design | Systematic Reviews | Primary studies  Scoping Reviews  Umbrella Reviews  Protocols |
| Population | Independently living, community-dwelling people 60+ living in single or multi-person households | People  who are employed,  have specific pre-existing conditions,  belong to a specific subgroup, e.g. veterans, immigrants, caregivers |
| Intervention | Preventive or health-promoting interventions: group format, delivered by peers or multipliers | Case managements  Exclusively online-based interventions without face-to-face contact  Pharmacological interventions  Animal-assisted interventions  Telehealth |
| Comparison | Any | --- |
| Outcomes | Any measures of social isolation and loneliness, social capital, social participation, social support and social networks |  |
| Setting | Community | Long-term care homes/facilities  Nursing homes |
