# Supplementary material for: The effectiveness of interventions to prevent loneliness and social isolation in the community-dwelling and old population: an overview of systematic reviews and meta-analysis
Source: Eur J Public Health. 2023 Mar 9;33(2):235–41. doi: 10.1093/eurpub/ckad006 (PMC10263264; doi:10.1093/eurpub/ckad006)
Supplement: ckad006_Supplementary_Data [file ckad006_Supplementary_Data.zip › ckad006_Supplementary_Data/ejph-2022-04-om-0208-File008.docx]

## Appendix D AMSTAR quality assessment

|  | AMSTAR 2 items ^a, c^ | | | | | | | | | | | | | | | | |
| --- | --- | --- | --- | --- | --- | --- | --- | --- | --- | --- | --- | --- | --- | --- | --- | --- | --- |
| Author, Year  [Reference] | 1 | 2^b^ | 3 | 4^b^ | 5 | 6 | 7^b^ | 8 | 9^b^ | 10 | 11^b^ | 12 | 13^b^ | 14 | 15^b^ | 16 | **Overall rating**(based on critical domains)**^d^** |
| Coll-Planas, 2017 | Y | Y* | N | Y* | Y | Y | Y | Y* | Y  * | N | 0 | 0 | Y | N | 0 | Y | moderate |
| Shvedko, 2018 | Y | Y | Y | Y* | Y | Y | Y | Y* | Y* | N | Y | 0 | Y | Y | Y | Y | moderate |
| Veazie, 2019 | Y | Y* | N | Y* | Y | Y | Y | Y | Y* | Y* | 0 | 0 | Y | N | 0 | Y | moderate |
| Peters, 2021 | Y | Y* | N | Y* | Y | Y | Y* | Y* | N | N | 0 | 0 | Y | N | 0 | Y | low |
| Poscia, 2018 | Y | N | N | Y* | Y | N | N | Y* | Y* | N | 0 | 0 | N | N | 0 | Y | Low |
| Li, 2018 | Y | N | Y | Y* | N | Y | Y | Y* | Y* | N | 0 | 0 | N | N | 0 | Y | low |
| Heins, 2021 | Y | Y | N | Y* | Y | Y | Y* | Y* | Y* | N | 0 | 0 | Y | Y | 0 | Y | moderate |
| Manjunath, 2021 | N | N | N | N | N | N | N | N | Y* | N | 0 | 0 | Y | N | 0 | Y | Critically low |
| Tong, 2021 | Y | N | N | N | Y | N | N | Y* | Y | N | 0 | 0 | N | Y | 0 | Y | Critically low |

*partial

^a^ Yes, No, Other

^b^ Critical Domains

^c^ AMSTAR 2 items:

1. **Did the research questions and inclusion criteria for the review include the components of PICO (Population, Intervention, Comparator group, Outcome)?** YES/NO. For yes, must have all four.
2. **Did the report of the review contain an explicit statement that the review methods were established prior to the conduct of the review and did the report justify any significant deviations from the protocol?** YES, PARTIAL YES, NO. For Partial YES: the authors state that they had a written protocol or guide that included ALL the following (review question(s), a search strategy, inclusion/exclusion criteria, a risk of bias assessment). For YES: as for partial yes, plus the protocol should be registered and should also have specified: a meta-analysis/synthesis plan, if appropriate, and a plan for investigating causes of heterogeneity, justification for any deviations from the protocol.
3. **Did the review authors explain their selection of the study designs for inclusion in the review?** YES/NO. For YES, the review should satisfy one of the following: explanation for including only RCTs, or explanation for including only NRSI, or explanation for including both RCTs and NRSI.
4. **Did the review authors use a comprehensive literature search strategy?** YES, PARTIAL YES, NO. for PARTIAL YES must have all of the following: searched at least 2 databases (relevant to research question), provided key word and/or search strategy, justified publication restrictions (eg. Language). For YES should also have all of the following: searched the reference lists/biographies of included studies, searched trial/study registries, included/consulted content experts in the field, searched for grey literature where relevant, conducted search within 24 months of completion of the review.
5. **Did the review authors perform study selection in duplicate?** YES/NO. for YES, either ONE of the following: at least two reviewers independently agreed on selection of eligible studies and achieved consensus on which studies to include OR two reviewers selected a sample of eligible studies and achieved good agreement (at least 80 per cent) with the remainder selected by one reviewer.
6. **Did the review authors perform data extraction in duplicate?** YES/NO. For YES, either one of the following: at least two reviewers achieved consensus on which data to extract from included studies OR two reviewers extracted data from a sample of eligible studies and achieved good agreement (at least 80 per cent) with the remainder extracted by one reviewer.
7. **Did the review authors provide a list of excluded studies to justify the exclusions?** YES, PARTIAL YES, NO. FOR partial yes must provide a list of all potentially relevant studies that were read in full text form but excluded from the review. For YES must also have justified the exclusion from the review of each potentially relevant study.
8. **Did the review authors describe the included studies in adequate detail?** YES, PARTIAL YES, NO. For PARTIAL YES, must describe all of the following: populations, interventions, comparators, outcomes, research designs. For YES should also have all of the following: described populations in detail, described intervention and comparator in detail (including doses where relevant), described study setting, timeframe or follow-up.
9. **Did the review authors use a satisfactory technique for assessing the risk of bias (RoB) in individual studies that were included in the review? For RCTs**: YES, PARTIAL YES, NO, INCLUDES ONLY NRSI. For PARTIAL YES must have assessed RoB from unconcealed allocation and lack of blinding of patients and assessors when assessing outcomes (unnecessary for objective outcomes such as all cause mortality); for YES must also have assessed RoB from allocation sequence that was not truly random and selection of the reported result from among multiple measurements or analyses of a specified outcome. **For NRSI** (Non Randomized Studies of Intervention)**:** YES, PARTIAL YES, NO, INCLUDES ONLY RCTs. For PARTIAL YES must have assessed RoB from confounding and from selection bias. For YES, must also have assessed methods used to ascertain exposures and outcomes, and selection of the reported results from among multiple measurements or analyses of a specified outcome.
10. **Did the review authors report on the sources of funding for the studies included in the review?** YES/NO. For YES: must have reported on the sources of funding for individual studies included in the review. Note: reporting that the reviewers looked for this information but it was not reported by study authors also qualifies
11. **If meta-analysis was performed, did the review authors use appropriate methods for statistical combination of results? For RCTs:** YES, NO, NO META-ANALYSIS. For YES: the authors justified combining the data in a meta-analysis and they used an appropriate weighted technique to combine study results and adjusted for heterogeneity if present and investigated the causes of heterogeneity. **For NRSI:** YES, NO, NO META-ANALYSIS CONDUCTED. For YES: the authors justified combining the data in a meta-analysis and they used an appropriate weighted technique to combine study results, adjusting for heterogeneity if present, and they statistically combined effects estimates from NRSI that were adjusted for confounding, rather than combining raw data, or justified combining raw data when adjusted effect estimates were not available, and they reported separate summary estimates for RCTs and NRSI separately when both were included in the review.
12. **If meta-analysis was performed, did the review authors assess the potential impact of RoB in individual studies on the results of the meta-analysis or other evidence synthesis?** YES, NO, NO META-ANALYSIS INCLUDED. For YES: included only low risk of bias RCTs or, if the pooled estimate was based on RCTs and/or NRSI at variable RoB, the authors performed analysis ton investigate possible impact of RoB on summary estimates of effect.
13. **Did the review authors account for RoB in individual studies when interpreting/discussing the results of the review?** YES/NO. for YES: included only low risk of bias RCTs or, if RCTs with moderate or high RoB, or NRSI were included, the review provided a discussion of the key impact of RoB on the results
14. **Did the review authors provide a satisfactory explanation for, and discussion of, any heterogeneity observed in the results of the review?** YES/NO. For Yes: there was no significant heterogeneity in the results OR if heterogeneity was present the authors performed an investigation of sources of any heterogeneity in the results and discussed the impact of this on the results of the review
15. **If they performed quantitative synthesis did the review authors carry out an adequate investigation of publication bias (small study bias) and discuss its likely impact on the results of the review?** YES, NO, NO META-ANALYSIS CONDUCTED. For YES: performed graphical statistical tests for publication bias and discussed the likelihood and magnitude of impact of publication bias
16. **Did the review authors report any potential sources of conflict of interest, including any funding they received for conducting the review?** YES/NO. For Yes: the authors reported no competing interests OR the authors described their funding sources and how they managed potential conflicts of interest.

**^d^** Rating overall confidence in the results of the review:

HIGH: *no on one non-critical weakness*: the systematic review provides an accurate and comprehensive summary of the results of the available studies that address the question of interest

MODERATE: *more than one non critical weakness* (multiple non-critical weaknesses may diminish confidence in the review and it may be appropriate to move the overall appraisal down from moderate to low confidence): the systematic review has more than one weakness but no critical flaws. It may provide an accurate summary of the results of the available studies that were included in the review

LOW: *one critical flaw with or without non-critical weaknesses*: the review has a critical flaw and may not provide an accurate and comprehensive summary of the available studies that address the question of interest

CRITICALLY LOW: *more than one critical flaw with or without non-critical weaknesses*: the review has more than one critical flaw and should not be relied on to provide an accurate and comprehensive summary of the available studies
