# Supplementary material for: The effectiveness of interventions to prevent loneliness and social isolation in the community-dwelling and old population: an overview of systematic reviews and meta-analysis
Source: Eur J Public Health. 2023 Mar 9;33(2):235–41. doi: 10.1093/eurpub/ckad006 (PMC10263264; doi:10.1093/eurpub/ckad006)
Supplement: ckad006_Supplementary_Data [file ckad006_Supplementary_Data.zip › ckad006_Supplementary_Data/ejph-2022-04-om-0208-File011.docx]

Appendix G Loneliness outcome: characteristics of studies with a low or moderate risk of bias.

| Type of Intervention (Effect on Loneliness) | Study Information | Type/Format of Intervention | Description of Intervention | Dose of Intervention | Staff |
| --- | --- | --- | --- | --- | --- |
| Alaviani 2015 ^38^  (+) | nRCT  N = 150  Mean age: NR  Moderate RoB  Iran | S/group | Multi-strategy programme based on constructs of Pender's Health  Promotion Model | (D) 10  (F) NR  (H) NR | NR |
| Chan 2017 ^42^  (+) | RCT  N = 48  Mean age: NR  Moderate RoB  China | M/group | Tai Chi Qigong with the assistance of elderly neighbourhood volunteers in strengthening social networks | (D) 3  (F) 2  (H) 1 | Professional (instructor, health ambassador) |
| Jung 2009 ^54^  (+) | nRCT  N = 45  Mean age: 66  Moderate RoB  Singapore | M/group | Combination of digital gaming and physical exercise | (D) 6  (F) 3  (H) NR | NR |
| Kahlbaugh 2011  ^55^  (+) | nRCT  N = 35  Mean age: 82  Moderate RoB  US | M/group | Combination of digital gaming and physical exercise | (D) 2.5  (F) 1  (H) NR | NR |
| Mountain 2017  ^59^  (+) | RCT  N = 288  Mean age: 72.1  Low RoB  UK | S/group | Facilitated group discussions on goal setting, sharing strengths and skills, and encouragement | (D) 4  (F) 1  (H) NR | Professional (social care staff, occupational therapist) |
| Vanoh 2019^71^  (+) | RCT  N = 50  Mean age: 68 moderate RoB  Malaysia | M/mixed | Web based health education intervention with group counselling | (D) 6  (F) 4  (H) 2 | NR |
| Dodge  2015  ^46^  (-) | RCT  N = 83  Mean age: 65  Moderate RoB  US | M/single | Social engagement in unstructured  conversation (i.e., conversation-based  cognitive stimulation) using personal  computers, webcams and user-friendly interactive Internet  communication programmes | (D) 1.5  (F) 7  (H) 3.5 | Professional (trained interviewers) |
| Pynnönen 2018  ^63^  (-) | RCT  N = 223  Mean age: 77  Moderate RoB  Finland | M/mixed | Choice of exercise, group discussions, day trips and art projects; or counselling | (D) 6  (F) 0.5  (H) 1.5 | Lay person and professional rehabilitation counsellor |
| Routasalo 2008  ^64^  (-) | RCT  N = 235  Mean age: 80  Low RoB  Finland | M/group | Psychosocial group rehabilitation with three types of activities: art and inspiring activities, group exercise and discussions, and therapeutic writing and group therapy | (D) 3  (F) 1  (H) 5 | Professional (nurse, occupational therapist) |
| Myhre  2017^70^  (-) | nRCT  N = 41  Mean age: 80  Moderate RoB  US | M/group | Using Facebook as an intervention to maintain or enhance cognitive function in older adults. | (D) 2  (F) NR  (H) NR | Professional (Instructor and tutors) |

(+) studies that had a positive effect; (-) studies that had no effect; RCT randomised controlled trial; nRCT non-randomised controlled trial; N Number of people in the study; NR not reported; RoB risk of bias; S social support intervention; M multicomponent intervention; (D) duration in months; (F) frequency per week; (H) hours per week.
