# Supplementary material for: The effectiveness of interventions to prevent loneliness and social isolation in the community-dwelling and old population: an overview of systematic reviews and meta-analysis
Source: Eur J Public Health. 2023 Mar 9;33(2):235–41. doi: 10.1093/eurpub/ckad006 (PMC10263264; doi:10.1093/eurpub/ckad006)
Supplement: ckad006_Supplementary_Data [file ckad006_Supplementary_Data.zip › ckad006_Supplementary_Data/ejph-2022-04-om-0208-File012.docx]

## Appendix H Social isolation/support outcome: characteristics of studies with a low or moderate risk for bias.

| Type of Intervention (Effect on Social Support) | Study Information | Type/Format of Intervention | Description of Intervention | Duration in Months, Intensity in Hours per Week | Staffing Requirements |
| --- | --- | --- | --- | --- | --- |
| Chan 2010  ^46^  (-) | RCT  N = 103  Mean age: 72  low RoB  China | P/mixed | Tai Chi Qigong group Exercise group (various breathing + walking exercises) | (D) 3  (F) 2  (H) 2 | Professional (non-medical healthcare provider) |
| Chan 2017  ^45^  (-) | RCT  N = 46  Mean age: 77  Moderate RoB  China | M/group | Tai Chi exercises + social support | (D) 3  (F) 2  (H) 2 | Professional (non-medical healthcare provider) |
| Huang 2011  ^53^  (-) | RCT  N = 93  Mean age: NR  low RoB  China | M/group | Cognitive behavioural therapy + Tai Chi exercises | (D) 2  (F) 5  (H) 1 | Professional (non-medical healthcare provider) |
| Lliffe 2014  ^54^  (-) | RCT  N = 183  Mean age: 73  low RoB  UK | P/group | Community-based group-exercise programme and home exercises: walking exercise, flexibility training, adapted Tai Chi complex, functional  skills training | (D) 6  (F) 2  (H) 1.5 | Professional (non-medical healthcare provider) |
| Imayama 2011  ^55^  (-) | RCT  N = 161  Mean age: 58  Low RoB  US | M/group | Dietary weight-loss group + aerobic exercise group | (D) 12  (F) 5  (H) 3.8 | Professional (medical healthcare provider) |
| Maki 2012 ^61^  (-) | RCT  N = 150  Mean age: NR  Low RoB  Japan | M/group | Exercise + health education: walking exercise | (D) 3  (F) 1  (H) 3 | Professional (non-medical healthcare provider) |
| Routasalo 2008  ^67^  (-) | RCT  N = 235  Mean age: 80  Low RoB  Finland | M/group | Psychosocial group rehabilitation with three types of activities: art and inspiring activities, group exercise and discussions, and therapeutic writing and group therapy | (D) 3  (F) 1  (H) 5 | Professional (nurse, occupational therapist) |
| Yap 2017 ^70^  (-) | RCT  N = 31  Mean age: 75  Moderate RoB | A/group | Rhythm-centred music making | (D) 3  (F) 1  (H) 1 | Professional (experienced instructors) |
| Vanoh 2019^74^  (-) | RCT  N = 50  Mean age: 68 moderate RoB  Malaysia | M/mixed | Web based health education intervention with group counselling | (D) 6  (F) 4  (H) 2 | NR |

(+) studies that had a positive effect; (-) studies that had no effect; RCT randomised controlled trial; N Number of people in the study; NR not reported; RoB risk of bias; A art intervention; M multicomponent intervention; P physical activity intervention; (D) duration in months; (F) frequency per week; (H) hours per week.
