# Supplementary material for: The effectiveness of interventions to prevent loneliness and social isolation in the community-dwelling and old population: an overview of systematic reviews and meta-analysis
Source: Eur J Public Health. 2023 Mar 9;33(2):235–41. doi: 10.1093/eurpub/ckad006 (PMC10263264; doi:10.1093/eurpub/ckad006)
Supplement: ckad006_Supplementary_Data [file ckad006_Supplementary_Data.zip › ckad006_Supplementary_Data/ejph-2022-04-om-0208-File013.docx]

## Appendix I Meta-analysis

Dodge (2015) did not provide results for follow-up but a regression analysis. We used the slope of the regression for Loneliness to approximate the difference at follow-up (e.g. 4.3–0.21). We used the pooled baseline standard deviation as a basis for our analysis.

Kahlbaugh (2011) exclusively reported follow-up results graphically. We extracted the results from the plot and used the baseline standard deviation. Further, the results of the treatment groups were quite different (watching TV vs. playing Wii games). The Wii games group was the only group that showed signs of a positive effect on loneliness.

Routasalo (2008) did not report follow-up outcomes but changes and their confidence intervals for social support. We used the changes as mean values for follow-up and approximated the standard deviation from the confidence intervals. Loneliness was only displayed graphically, with no recognisable changes from baseline. The authors reported, ‘However, there were no differences in the changes in the UCLA-scale between the groups at 3 or 6 months’. The standard deviation for loneliness was missing. Hence, we used the standard deviation for UCLA from a large panel survey: https://journals.sagepub.com/doi/full/10.1177/0890117119856551

Jung (2009) used the UCLA scale and reported very low mean values and standard deviations. It is unclear why these values are so low. The authors did not provide information on data transformation.

Yap (2017), Imayama (2011), Myrhe (2017), Engelbrecht (2015), and Pynnönen (2018) provided insufficient data for MA.

We combined the CB and CB + Tai Chi groups for Huang (2011). We combined the exercise and Tai Chi groups for Chan (2010) and Chan (2017). We combined the FaME and OEP groups for Iliffe (2014).

In case the studies used multiple tools to measure a single construct, we only used the most regular reported outcomes (e.g. UCLA or Lubben Social Network Scale).
