# Supplementary material for: The effectiveness of interventions to prevent loneliness and social isolation in the community-dwelling and old population: an overview of systematic reviews and meta-analysis
Source: Eur J Public Health. 2023 Mar 9;33(2):235–41. doi: 10.1093/eurpub/ckad006 (PMC10263264; doi:10.1093/eurpub/ckad006)
Supplement: ckad006_Supplementary_Data [file ckad006_Supplementary_Data.zip › ckad006_Supplementary_Data/ejph-2022-04-om-0208-File014.docx]

Appendix J additional References

Additional References

38 Alaviani M, Khosravan S, Alami A, Moshki M. The Effect of a Multi-Strategy Program on Developing Social Behaviors Based on Pender’s Health Promotion Model to Prevent Loneliness of Old Women Referred to Gonabad Urban Health Centers. International Journal of Community Based Nursing and Midwifery 2015;3:132-140.

39 Andersson L. Intervention against loneliness in a group of elderly women: an impact evaluation. Social science & medicine (1982) 1985;20:355-364.

40 Bartlett H, Warburton J, Lui C-W, Peach L, Carroll M. Preventing social isolation in later life: findings and insights from a pilot Queensland intervention study. Ageing and Society 2012;33:1167-1189.

41 Bøen H, Dalgard OS, Johansen R, Nord E. A randomized controlled trial of a senior centre group programme for increasing social support and preventing depression in elderly people living at home in Norway. BMC geriatr 2012;12:20.

42 Chan AW, Yu DS, Choi KC. Effects of tai chi qigong on psychosocial well-being among hidden elderly, using elderly neighborhood volunteer approach: a pilot randomized controlled trial. Clin Interv Aging 2017;12:85-96.

43 Chan AWK, Lee A, Suen LKP, Tam WWS. Effectiveness of a Tai chi Qigong program in promoting health-related quality of life and perceived social support in chronic obstructive pulmonary disease clients. Quality of Life Research 2010;19:653-664.

44 Coll-Planas L, Del Valle Gómez G, Bonilla P, Masat T, Puig T, Monteserin R. Promoting social capital to alleviate loneliness and improve health among older people in Spain. Health & social care in the community 2017;25:145-157.

45 Davidson JW, McNamara B, Rosenwax L, Lange A, Jenkins S, Lewin G. Evaluating the potential of group singing to enhance the well-being of older people. Australasian Journal on Ageing 2014;33:99-104.

46 Dodge HH, Zhu J, Mattek NC, et al. Web-enabled conversational interactions as a method to improve cognitive functions: Results of a 6-week randomized controlled trial. Alzheimer's & Dementia: Translational Research & Clinical Interventions 2015;1:1-12.

47 Gonyea JG, Burnes K. Aging Well at Home: Evaluation of a Neighborhood-based Pilot Project to “Put Connection Back into Community”. Journal of Housing For the Elderly 2013;27:333-347.

48 Hind D, Mountain G, Gossage-Worrall R, et al. Putting Life in Years (PLINY): a randomised controlled trial and mixed-methods process evaluation of a telephone friendship intervention to improve mental well-being in independently living older people. Southampton (UK); 2014.

49 Honigh - de Vlaming R. Healthy Ageing: Prevention of Loneliness among Elderly People : Evaluation of a Complex Intervention in Public Health Practice [Ph.D.]. Wageningen University and Research, 2013.

50 Huang TT, Yang LH, Liu CY. Reducing the fear of falling among community-dwelling elderly adults through cognitive-behavioural strategies and intense Tai Chi exercise: a randomized controlled trial. Journal of advanced nursing 2011;67:961-971.

51 Iliffe S, Kendrick D, Morris R, et al. Multicentre cluster randomised trial comparing a community group exercise programme and home-based exercise with usual care for people aged 65 years and over in primary care. Health technology assessment (Winchester, England) 2014;18:vii-xxvii, 1-105.

52 Imayama I, Alfano CM, Kong A, et al. Dietary weight loss and exercise interventions effects on quality of life in overweight/obese postmenopausal women: a randomized controlled trial. Int J Behav Nutr Phys Act 2011;8:118.

53 Jones RB, Ashurst EJ, Atkey J, Duffy B. Older people going online: its value and before-after evaluation of volunteer support. J Med Internet Res 2015;17:e122.

54 Jung Y, Li KJ, Janissa NS, Gladys WLC, Lee KM. Games for a better life. Proceedings of the Proceedings of the Sixth Australasian Conference on Interactive Entertainment - IE '09; 2009 Sydney, Australia: 2009:1-6.

55 Kahlbaugh PE, Sperandio AJ, Carlson AL, Hauselt J. Effects of Playing Wii on Well-Being in the Elderly: Physical Activity, Loneliness, and Mood. Activities, Adaptation & Aging 2011;35:331-344.

56 Kamegaya T, Araki Y, Kigure H, Yamaguchi H. Twelve-week physical and leisure activity programme improved cognitive function in community-dwelling elderly subjects: a randomized controlled trial. Psychogeriatrics 2014;14:47-54.

57 Low LF, Baker JR, Harrison F, et al. The Lifestyle Engagement Activity Program (LEAP): Implementing Social and Recreational Activity into Case-Managed Home Care. J Am Med Dir Assoc 2015;16:1069-1076.

58 Maki Y, Ura C, Yamaguchi T, et al. Effects of intervention using a community-based walking program for prevention of mental decline: a randomized controlled trial. J Am Geriatr Soc 2012;60:505-510.

59 Mountain G, Windle G, Hind D, et al. A preventative lifestyle intervention for older adults (lifestyle matters): a randomised controlled trial. Age Ageing 2017;46:627-634.

60 Nicholson NR, Jr., Shellman J. Decreasing social isolation in older adults: effects of an empowerment intervention offered through the CARELINK program. Res Gerontol Nurs 2013;6:89-97.

61 Perkins P. Impact of a horticultural therapy program on the well-being of low-income community dwelling older adults. International Society for Horticultural Science (ISHS), Leuven, Belgium, 2012:123-132.

62 Phinney A, Moody EM, Small JA. The Effect of a Community-Engaged Arts Program on Older Adults' Well-being. Can J Aging 2014;33:336-345.

63 Pynnönen K, Törmäkangas T, Rantanen T, Tiikkainen P, Kallinen M. Effect of a social intervention of choice vs. control on depressive symptoms, melancholy, feeling of loneliness, and perceived togetherness in older Finnish people: a randomized controlled trial. Aging Ment Health 2018;22:77-84.

64 Routasalo PE, Tilvis RS, Kautiainen H, Pitkala KH. Effects of psychosocial group rehabilitation on social functioning, loneliness and well-being of lonely, older people: randomized controlled trial. Journal of advanced nursing 2009;65:297-305.

65 Saito T, Kai I, Takizawa A. Effects of a program to prevent social isolation on loneliness, depression, and subjective well-being of older adults: A randomized trial among older migrants in Japan. Archives of Gerontology and Geriatrics 2012;55:539-547.

66 Xu X, Li J, Pham TP, Salmon CT, Theng Y-L. Improving Psychosocial Well-Being of Older Adults Through Exergaming: The Moderation Effects of Intergenerational Communication and Age Cohorts. Games for Health Journal 2016;5:389-397.

67 Yap AF, Kwan YH, Tan CS, Ibrahim S, Ang SB. Rhythm-centred music making in community living elderly: a randomized pilot study. BMC Complement Altern Med 2017;17:311.

68 Matz-Costa C, Lubben J, Lachman ME, Lee H, Choi YJ. A Pilot Randomized Trial of an Intervention to Enhance the Health-Promoting Effects of Older Adults' Activity Portfolios: The Engaged4Life Program. J Gerontol Soc Work 2018;61:792-816.

69 Lee OE-K, Kim D-H. Bridging the Digital Divide for Older Adults via Intergenerational Mentor-Up. Research on Social Work Practice 2018;29:786-795.

70 Myhre JW, Mehl MR, Glisky EL. Cognitive Benefits of Online Social Networking for Healthy Older Adults. J Gerontol B Psychol Sci Soc Sci 2017;72:752-760.

71 Vanoh D, Shahar S, Razali R, et al. The Effectiveness of a Web-Based Health Education Tool, WESIHAT 2.0, among Older Adults: A Randomized Controlled Trial. J Alzheimers Dis 2019;70:S255-S270.

72 Ware C, Damnee S, Djabelkhir L, et al. Maintaining Cognitive Functioning in Healthy Seniors with a Technology-Based Foreign Language Program: A Pilot Feasibility Study. Front Aging Neurosci 2017;9:42.

73 Engelbrecht R, Shoemark H. The Acceptability and Efficacy of Using iPads in Music Therapy to Support Wellbeing with Older Adults: A Pilot Study. The Australian Journal of Music Therapy 2015;26:49-70.

74 De Jong Gierveld J, Van Tilburg T. The De Jong Gierveld short scales for emotional and social loneliness: tested on data from 7 countries in the UN generations and gender surveys. European Journal of Ageing 2010;7:121-130.

75 Russell DW. UCLA Loneliness Scale (Version 3): Reliability, Validity, and Factor Structure. Journal of Personality Assessment 1996;66:20-40.

76 Hughes ME, Waite LJ, Hawkley LC, Cacioppo JT. A Short Scale for Measuring Loneliness in Large Surveys. Research on Aging 2004;26:655-672.

77 Barrera M, Sandler IN, Ramsay TB. Preliminary development of a scale of social support: Studies on college students. American Journal of Community Psychology 1981;9:435-447.

78 Lubben J, Blozik E, Gillmann G, et al. Performance of an Abbreviated Version of the Lubben Social Network Scale Among Three European Community-Dwelling Older Adult Populations. The Gerontologist 2006;46:503-513.

79 Dahlem NW, Zimet GD, Walker RR. The Multidimensional Scale of Perceived Social Support: a confirmation study. J Clin Psychol 1991;47:756-761.

80 Kocalevent R-D, Berg L, Beutel ME, et al. Social support in the general population: standardization of the Oslo social support scale (OSSS-3). BMC Psychology 2018;6.

81 Chou K-L. Assessing Chinese adolescents’ social support: the multidimensional scale of perceived social support. Personality and Individual Differences 2000;28:299-307.

82 Kempen GIJM, Van Eijk LM. The Psychometric Properties of the SSL12-I, a Short Scale for Measuring Social Support in the Elderly. Social Indicators Research 1995;35:303-312.

83 Sherbourne CD, Stewart AL. The MOS social support survey. Social science & medicine (1982) 1991;32:705-714.

84 Cornwell EY, Waite LJ. Measuring Social Isolation Among Older Adults Using Multiple Indicators From the NSHAP Study. The Journals of Gerontology Series B: Psychological Sciences and Social Sciences 2009;64B:i38-i46.

85 Hawthorne G, Griffith P. The Friendship Scale: Development and properties. 2017.

# 
